# Occurrence and Characteristics of Microplastics in Raw Milk from Smallholder Dairy Farms in Northeastern Thailand

**DOI:** 10.3390/ani16030409

**Published:** 2026-01-28

**Authors:** Penkhae Thamsenanupap, Suksan Bunkrachang, Chayanis Paenlam, Sireethon Dejboonchuai, Tawatchai Tanee, Piemjit Muangkot, Warut Donrung, Natapol Pumipuntu

**Affiliations:** 1 One Health Research Unit, Mahasarakham University, Maha Sarakham 44000, Thailand; penkhae.t@msu.ac.th (P.T.); 2543warut@gmail.com (W.D.); 2 Faculty of Environment and Resource Studies, Mahasarakham University, Maha Sarakham 44150, Thailand; 3 Faculty of Veterinary Sciences, Mahasarakham University, Maha Sarakham 44000, Thailand

**Keywords:** microplastics, raw milk, smallholder daily farm, raw milk production

## Abstract

Microplastic contamination in food systems has emerged as a growing concern for food safety and public health. The presence of microplastics in raw milk may represent a potential exposure pathway for both animals and humans. This study investigated the occurrence and characteristics of microplastics in raw milk collected from smallholder dairy farms in Northeastern Thailand. Hand-milked raw milk and bulk tank milk samples were obtained from ten farms and analyzed for microplastic contamination. Suspected microplastic particles were identified and quantified using stereomicroscopy and characterized according to their shape, color, and size, while polymer composition was confirmed by Fourier Transform Infrared Spectroscopy. Microplastics were detected in both hand-milked and bulk tank milk samples, with fiber-shaped particles being the most frequently observed. The majority of detected particles were 0.05–0.15 mm in size and predominantly yellow in color. Polymer analysis revealed that Polydimethylsiloxane, followed by a semi-synthetic composite of Elastane and Rayon. These findings demonstrate that microplastics can be present in raw milk produced by smallholder dairy farms, highlighting the need for improved farm management and milk-handling practices to reduce contamination risks. From a One Health perspective, reducing plastic use and enhancing hygiene during milking and milk storage may help protect animal health, food safety, and consumer well-being.

## Simple Summary

Plastic waste can gradually break down into very small particles known as microplastics, which may enter food products consumed by humans. This study examined the occurrence of microplastics in raw milk from smallholder dairy farms in Maha Sarakham Province, northeastern Thailand. Samples of hand-milked raw milk and bulk tank milk were collected from ten farms and analyzed for microplastic contamination. Microplastics were detected in both types of milk samples, with fiber-shaped particles being the most common form. Most particles were yellow in color, suggesting potential sources related to synthetic materials used during farm activities or milk handling. The predominant polymers identified were Polydimethylsiloxane, followed by a semi-synthetic composite of Elastane and Rayon. These findings indicate that microplastics can be present in raw milk produced by smallholder dairy farms, raising concerns for food safety and public health. From a One Health perspective, improving farm hygiene practices, reducing contact with plastic materials, and strengthening management during milking and milk storage may help lower the risk of microplastic contamination in dairy products.

## 1. Introduction

Plastic is a synthetic and semi-synthetic material composed of polymers and a variety of chemical additives, including colorants, plasticizers, and stabilizers [1]. Since it was first synthesized in 1907, plastic has played a transformative role across numerous sectors, notably in healthcare, agriculture, and the food industry [2]. Its affordability, functional versatility, and long-term durability have contributed to its widespread and growing utilization. Consequently, the rate of global plastic production has surged and is expected to accelerate further in the future [3]. Nonetheless, the rapid expansion of plastic production and use has led to escalating environmental concern. This issue is characterized by the persistent accumulation of plastic waste and widespread microplastic contamination across ecological systems and the food chain [4].

Microplastics (MPs) are defined as synthetic or semi-synthetic plastic particles ranging in size less than 5000 µm [5]. They originate from two principal sources: primary MPs, which are deliberately manufactured for use in products such as cosmetics and personal care items, and secondary MPs, which arise from the environmental degradation of larger plastic debris [6]. Both categories of MPs pose substantial challenges to environmental health due to their resistance to natural decomposition processes. Their persistent accumulation across diverse ecosystems poses serious risks to wildlife and domestic animal populations [7]. This widespread contamination also raises significant concerns for human health [4], which underscores a critical challenge within the One Health framework.

The widespread occurrence of MPs in the environment has emerged as a significant public health and food safety concern. Recent investigations have confirmed that microplastic particles are not limited to marine and freshwater environments. They are also prevalent in the soil, plants, and atmosphere, which affect various aspects of daily life for both humans and animals [8,9,10,11]. Although global awareness of microplastic pollution is increasing, significant knowledge gaps remain concerning their sources, levels of distribution, and potential health impacts through the food chain.

Milk is an important dietary component that provides essential nutrients such as calcium, high-quality protein, and beneficial fats [12]. Global production and consumption of milk have steadily increased in recent years [13]. Among major food groups, dairy products serve as an important source of nutrients for both humans and animals. Despite their dietary importance, microplastic contamination in milk has not been widely investigated, especially in Thailand. This research gap concerns given the potential for MPs to enter the milk production chain. The presence of MPs in dairy products may pose health risks to consumers and livestock, underscoring the need for more focused scientific attention in this area [6]. As awareness of this emerging issue, our present study aims to investigate microplastic contamination in milk collected from smallholder dairy farms in the northeastern part of Thailand, an important region for national dairy production [14]. This study aimed to investigate the occurrence, physical characteristics, and polymer composition of semi-synthetic and synthetic MPs in raw milk from smallholder dairy farms in northeastern Thailand.

## 2. Materials and Methods

### 2.1. Ethics Statement

This research project was approved and conducted with the animal-use protocol approved by the Institutional Animal Care and Use Committee (IACUC) of Mahasarakham University (protocol numbers: IACUC-MSU-024-011/2025). 

### 2.2. Study Area

This cross-sectional study was conducted in a dairy farming area in Northeastern Thailand, a region characterized by smallholder dairy production. A field survey identified ten operational smallholder dairy farms for inclusion in the study. All selected farms were located within the same agroecological setting and shared comparable management practices, including the use of Holstein Friesian cattle and a single-bucket milking system for raw milk collection. Milk from individual cows was collected by hand milking to assess microplastic presence at the animal level, while bulk tank milk was sampled to represent pooled milk at the farm level, as shown in Table 1. Although minor differences existed among farms in terms of herd size and daily milk yield, overall management practices related to milking and milk handling were broadly similar.

### 2.3. Sample Collection

The sample set for this study consisted of 70 hand-milked raw milk samples (15 mL per sample), each obtained by pooling milk from all four teats of individual lactating cows. In addition, 10 bulk tank milk samples (one sample per farm; 15 mL per sample) were collected to represent pooled milk at the farm level. All milk samples were immediately transported to the One Health Research Unit laboratory, Mahasarakham University, under chilled conditions for further processing and analysis. Throughout the sampling procedure, strict measures were taken to minimize external contamination, including the exclusive use of non-plastic collection tools and containers.

### 2.4. Sample Processing

For laboratory preparation, raw milk samples (both hand-milked and bulk tank) were set at room temperature prior to analysis. A volume of 15 mL from each sample was transferred into a clean glass Erlenmeyer flask, followed by the addition of 15 mL of 30% hydrogen peroxide solution (H_2_O_2_) for 12 h followed Thamsenanupap et al. [8]. The mixtures were then subjected to oxidative digestion by incubation in a temperature-controlled orbital shaker at 200 revolutions per minute (rpm) and 50 °C for one hour, to facilitate the breakdown of organic material. After digestion, the samples were filtered using 47 mm diameter, 1.2 μm pore-size microfiber filter paper (Whatman GF/C, Cytiva, Wilmington, DE, USA). The filters were placed in sterile 90 mm glass Petri dishes and subsequently dried in a hot air oven at 60 °C for 24 h. Once dried, the filters were set to room temperature and stored in covered Petri dishes until further microscopic examination and microplastic analysis.

To minimize external contamination during microplastic extraction and characterization, all sample processing was conducted under controlled clean-air laboratory conditions. Prior to use, glassware was rinsed three times with filtered distilled water to remove potential residual particles, and researchers wore cotton lab coats to reduce synthetic fiber shedding. Blank control samples were incorporated at every analytical step to monitor potential airborne contamination during handling and filtration. In addition, a negative control for microplastic analysis was performed by subjecting a reagent solution to the same digestion protocol used for actual samples, including identical chemicals, glassware, and filtration procedures, but without any organic or biological material. The resulting blank sample was filtered and examined to detect and quantify any microplastic particles originating from laboratory reagents, equipment, or handling processes.

### 2.5. Analysis

Microplastic particles were meticulously observed and analyzed using a stereomicroscope (Zeiss Stemi 305 Standard K-Lab, Carl Zeiss Microscopy GmbH, Jena, Germany). Initial identification of MPs abundance, color, and morphology (categorized as fiber, fragment, pellet, or foam) relied on visual characterization consistent with criteria informed by Thamsenanupap et al. [8]. This initial screening approach is crucial in veterinary public health investigations to assess potential MP contamination. To confirm the identity of visually suspected microplastics, representative particles were selected for FT-IR spectroscopy using a stratified sampling approach across morphology, color, and size categories, consistent with established practices in microplastic research where representative subsampling is commonly applied [15,16]. Particles were first grouped by sample type (hand-milked raw milk and bulk tank milk) and then proportionally selected to represent the diversity of shape, size, and color observed. Rare or atypical particles were included to ensure representation of less common morphologies. When a subgroup contained few particles, at least one sample was analyzed. This strategy allowed verification of MPs across particle categories while maintaining analytical feasibility. These samples were subjected to Fourier-transform infrared spectroscopy (FTIR) utilizing the Attenuated Total Reflection (ATR) mode (PerkinElmer Frontier, Spotlight 200i model, PerkinElmer, Shelton, CT, USA). The resulting spectra from each potential MP were then rigorously compared against a comprehensive reference library. Only particles exhibiting spectral similarity exceeding 90% to a standard reference material were definitively confirmed and reported as MPs.

To minimize microplastic contamination during processing and analysis, we implemented a strict control regime. This included regular cleaning of the working area, performing all procedures within a laboratory fume hood, and ensuring personnel wore cotton laboratory coats. Furthermore, glassware was exclusively used over plastic to prevent potential MPs contamination from laboratory consumables.

### 2.6. Data Analysis

Descriptive statistics were performed to analyze the occurrence and concentration of MPs accumulation in raw milk, dairy calf manure, water samples, and feed from smallholder farms in Kantharawichai District, Maha Sarakham. Proportions and 95% confidence intervals were calculated to estimate the prevalence and distribution of microplastic particles across the sample groups. All statistical analyses were conducted using SPSS Statistics software (version 22, IBM Corp., Armonk, NY, USA).

## 3. Results

### 3.1. Abundance and Distribution of Microplastics in Raw Milk from Dairy Farms

A total of 80 milk samples were obtained from 10 smallholder dairy farms for microplastic analysis, yielding an overall count of 97 particles across two milk sample types. Microplastics were detected in both hand-milked raw milk and bulk tank milk samples. Hand-milked raw milk accounted for the majority of detected particles (70 particles), while 27 particles were identified in bulk tank milk samples. At the farm level, the number of microplastic particles detected in hand-milked raw milk ranged from 0 to 30 particles per farm, with no particles detected in samples from Farm C and Farm I. Bulk tank milk samples contained between 0 and 6 particles per farm; notably, no MPs were detected in bulk tank milk samples from Farm C and Farm H.

Overall, microplastic detection was consistent across farms and sample categories, with all sampled locations presenting identifiable levels of microplastic contamination. A detailed distribution of microplastic counts across sample types and farms is provided in Table 2.

### 3.2. Morphology, Color, and Size of Microplastics in Dairy Farms

**Figure 1:**
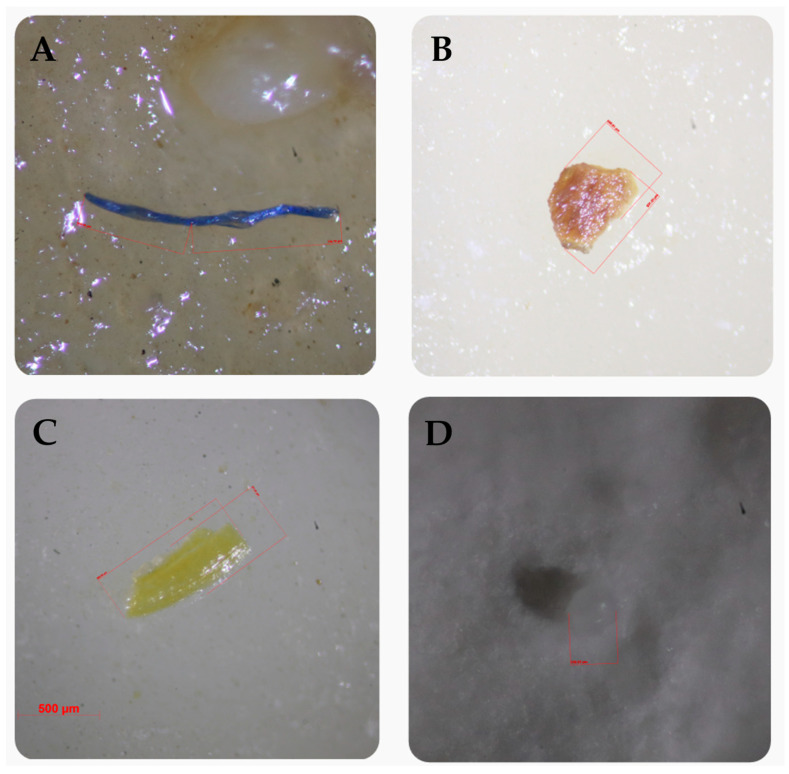
Morphological types of microplastics detected in dairy farm samples under a stereoscope. (**A**) fiber, (**B**) fragment, (**C**) film, and (**D**) microbead.

The morphological characteristics of MPs detected in hand-milked raw milk and bulk tank milk are summarized in Table 3 and demonstrated in Figure 1. A total of 97 microplastic particles were identified across both sample types, comprising 70 particles from hand-milked raw milk and 27 particles from bulk tank milk. Overall, fiber was the dominant morphology, accounting for more than half of all detected particles (52.6%). In hand-milked raw milk, fibers were the most prevalent form, representing 56.0% (39/70) of the particles, followed by fragments at 37.0% (26/70) and microbeads at 7.0% (5/70). In contrast, bulk tank milk showed a slightly different distribution, with fibers accounting for 44.0% (12/27) and fragments comprising 40.0% (11/27) of the detected particles, while microbeads constituted 11.0% (3/27). Notably, film-type MPs were only detected in bulk tank milk (4.0%, 1/27) and were absent from hand-milked raw milk. When combined, fibers (*n* = 51) and fragments (*n* = 37) constituted the majority of MPs detected across both sampling methods, indicating that elongated and irregularly shaped particles were the predominant forms present in raw milk samples.

The color distribution of MPs identified in hand-milked raw milk and bulk tank milk is also presented in Table 3. In hand-milked raw milk, yellow particles were the most frequently observed, accounting for 37.0% (26/70) of the detected MPs, followed by white particles at 27.0% (19/70). Black and blue particles were detected at similar proportions, each representing 13.0% (9/70), while red, brown, and green particles were present at relatively low frequencies (≤4.0%). In bulk tank milk, however, black MPs were the dominant color, comprising 45.0% (12/27) of the total particles, followed by white (22.0%, 6/27) and yellow (15.0%, 4/27). Brown and blue particles were less common, accounting for 11.0% and 7.0%, respectively, whereas red and green particles were not detected in bulk tank milk. When data from both sample types were combined, yellow (30.9%) and white (25.8%) particles were the most abundant overall, followed by black particles (21.6%). These findings demonstrate distinct differences in color profiles between hand-milked raw milk and bulk tank milk, suggesting potential differences in contamination sources or post-collection handling processes. The size distribution of MPs detected in hand-milked raw milk and bulk tank milk is detailed in Table 3. In hand-milked raw milk, all detected MPs (100%, 70/70) fell within the smallest size class (0.05–0.15 mm), indicating a highly uniform size distribution dominated by very small particles. In contrast, bulk tank milk exhibited a broader size range. Although the smallest size class (0.05–0.15 mm) remained predominant, accounting for 37.0% (10/27), a substantial proportion of particles fell within the 0.15–0.5 mm size range (40.7%, 11/27). Larger particles were also detected exclusively in bulk tank milk, including those sized >0.5–1 mm (14.8%, 4/27) and >1–2 mm (7.4%, 2/27). When combined across both sample types, MPs in the 0.05–0.15 mm size class accounted for 82.5% (80/97) of all detected particles, highlighting the predominance of small MPs in raw milk. The presence of larger size fractions solely in bulk tank milk suggests that aggregation processes or additional contamination may occur during milk collection, storage, or handling prior to sampling.

### 3.3. Fourier Transform Infrared (FT-IR) Spectroscopy for Microplastic Analysis

**Figure 2:**
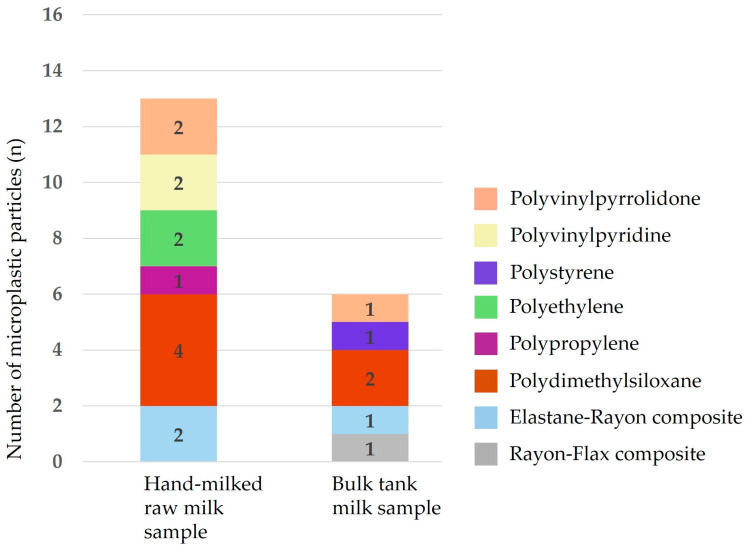
Distribution of microplastic polymer types by raw milk sample source.

Fourier Transform Infrared (FT-IR) spectroscopy was conducted to identify the polymer composition of selected suspected microplastic particles that were isolated from hand-milked raw milk and bulk tank milk samples, as shown in Figure 2. A total of 19 representative microplastic particles were successfully characterized, comprising 13 particles from hand-milked samples and 6 particles from bulk tank samples. Overall, polydimethylsiloxane was the most frequently identified polymer, accounting for 31.6% of the analyzed particles, with four particles detected in hand-milked samples and two in bulk tank samples. Polyvinylpyrrolidone, polyvinylpyridine, and polyethylene were each detected in 10.5% of the particles and were identified exclusively in hand-milked raw milk samples. A semi-synthetic elastane–rayon composite was detected in 15.8% of the analyzed particles, including two particles from hand-milked samples and one from bulk tank milk. Less frequently detected polymers included polypropylene, polystyrene, and a rayon–flax composite, each accounting for 5.3% of the total particles analyzed, with polypropylene identified in hand-milked samples, while polystyrene and rayon–flax composite were detected in bulk tank milk samples. The FT-IR results demonstrate the presence of multiple synthetic and semi-synthetic polymer types in both hand-milked raw milk and bulk tank milk samples.

## 4. Discussion

The detection of MPs in raw milk samples, including those collected directly through hand milking and from bulk tank storage, demonstrates that these particles are present within the milk produced by dairy cows. The identification of MPs in hand-milked raw milk indicates that microplastic particles are already present in milk at the point of secretion, suggesting internal exposure and potential translocation within the animal [17]. These findings of microplastic contamination in raw milk are consistent with previous studies describing microplastic occurrence and potential transmission pathways in livestock systems [17,18,19,20], which have reported the presence of MPs in milk produced by dairy cows, suggesting that microplastic exposure may occur at the animal level and be reflected in secreted milk [17]. To further contextualize our results quantitatively, the mean concentration of MPs in hand-milked raw milk in the present study was 0.057 ± 0.052 particles/mL, as demonstrated in Table 2, equivalent to approximately 5.7 ± 5.2 particles/100 mL. This level is markedly lower than concentrations reported in cow’s milk, where Da Costa Filho et al. (2021) [17] observed values ranging from 204 to 1004 particles per 100 mL. In addition, our findings are consistent with previous studies reporting the presence of MPs within dairy production systems. Investigations by Yoon et al. (2024) [11], Jahandari (2023) [21], and Cai et al. (2023) [22] have documented microplastic contamination in dairy farm–related matrices, suggesting that MPs can occur within dairy production environments and may ultimately be detected in raw milk.

The predominance of fiber-shaped MPs and the detection of yellow color imply that contamination may arise from synthetic fibers in clothing, feed bags, or equipment that is consistent with the observations reported by Pulikkoden et al. [23]. The observed variation in particle size and morphology may reflect degradation of plastic materials commonly used on farms, including those involved in feed preparation and packaging, storage covers, and milking-related equipment [24]. Differences in color and size distribution may also correspond to variation in materials and handling practices among farms. In addition, this size range was particularly dominant in hand-milked raw milk samples, aligning with the findings of Rbaibi Zipak et al. (2024) [25], who reported comparable microplastic dimensions in raw bovine milk.

The detection of small-sized MPs (0.05–0.15 mm) in hand-milked raw milk is highly relevant from a biological and toxicological perspective. Previous experimental and observational studies indicate that smaller MPs exhibit greater bioavailability, with enhanced capacity to cross biological barriers such as the intestinal epithelium through paracellular transport, M-cell uptake, or endocytosis [6]. In ruminants, repeated ingestion of MPs via contaminated feed may allow particles below 150 µm to translocate from the gastrointestinal tract into systemic circulation [17]. Once in the bloodstream, small MPs may reach peripheral organs, including the mammary gland, where they can potentially cross epithelial barriers and be excreted into milk. Similar size-dependent transfer mechanisms have been proposed in experimental models and human food contamination studies, where MPs < 150 µm were preferentially detected in milk and dairy products [17,25]. From a One Health perspective, the predominance of small MPs in milk is of particular concern. MPs in the 50–150 µm range are considered more hazardous to humans than larger particles due to their increased potential of interacting with intestinal epithelial cells and penetrating the intestinal epithelium after ingestion [4].

FT-IR analysis identified polydimethylsiloxane and a semi-synthetic composite material composed of elastane and rayon as the two most frequently detected polymer types among the analyzed representative microplastic particles found in raw milks. This result was different from findings by Corte Pause et al. (2024) [19], who reported Polyethylene and Polyvinylchloride as the dominant types in raw milk samples from dairy farms in the Marmara region of Turkey. The predominance of a semi-synthetic composite material such as Elastane combined with Rayon in this study may reflect contamination sources linked to textile-derived fibers, such as clothing worn by farm workers, cleaning cloths, and feed storage materials [26,27]. Elastane (also known as spandex or Lycra) is a synthetic polymer that is widely used in textile manufacturing due to its exceptional elasticity and strength [28]. Rayon is a semi-synthetic cellulose-based fiber produced by chemically processing natural polymers, typically derived from wood pulp. Although it originates from plant material, the manufacturing process involves intensive treatment with chemicals, resulting in a fiber that behaves similarly to synthetic textiles in terms of durability and environmental persistence [29]. This fiber composite material is lightweight and can easily become airborne. It can adhere to equipment or settle on feed and milk during handling, especially in open or semi-enclosed milking environments [30]. This semi-synthetic fabric used in clothing, cleaning clothes, and feed-storage materials can shed fine fibers through friction, washing, and repeated use. Once released, these microfibers behave like MPs and can enter the dairy environment, providing a possible pathway for contamination within smallholder dairy farms [28,29].

In addition, the presence of Polydimethylsiloxane (PDMS) might indicate that feed storage or preservation processes may serve as an additional source of synthetic polymer contamination within the dairy environment. PDMS, a silicone-based polymer, can be used as a coating agent on animal feed ingredients to extend shelf-life and reduce the effects of spoilage organisms and oxidative damage [31]. As coated feed is routinely mixed, stored, and handled within farm facilities, abrasion during transport or feeding may release fragments of PDMS into the surrounding environment. This mechanism suggests that preserved feed products represent another potential source of microplastic contamination within smallholder dairy systems [32].

In contrast, the polymer types reported by Corte Pause et al. (2024) [19] such as Polyethylene and Polyvinylchloride were the principal microplastic contaminants in raw milk samples collected from dairy farms in the Marmara region of Turkey. Previous research has also reported that these polymer types are commonly associated with packaging materials, plastic containers, and tubing frequently used in industrialized dairy operations [33]. The differences between studies may therefore be attributed to variations in agricultural materials, climatic conditions, and regional practices regarding plastic use and waste management [34,35]. For instance, smallholder farms in tropical climates, such as those in Northeastern Thailand, may experience higher airborne particle movement, fiber shedding from clothing, and accelerated degradation of plastics under heat and sunlight, all of which can influence contamination profiles [36].

These results emphasize the importance of improving farm hygiene, reducing dependency on synthetic materials, and managing plastic waste responsibly to minimize microplastic exposure in dairy environments. Targeted interventions, such as promoting natural fiber clothing for farm workers, using covered storage systems, and ensuring regular maintenance of milking equipment, could substantially reduce the risk of microplastic contamination [4,37]. Moreover, continuous monitoring of polymer types through FT-IR spectroscopy can help trace contamination sources and evaluate the effectiveness of mitigation strategies over time [20,38,39].

Nevertheless, this study has methodological limitations related to microplastic identification. Visually suspected particles were recorded, with a subset confirmed using FT-IR spectroscopy, following a commonly adopted workflow in microplastic research [40]. FT-IR spectroscopy is reliable for polymer identification of particles typically larger than 20 µm. However, its analytical performance is reduced for smaller particles due to inherent spatial resolution and spectral constraints. Accordingly, particles within the lower size range potentially captured by filtration were not included in size-based analyses, as reliable polymer confirmation for these particles would require complementary analytical techniques beyond FT-IR alone. Future studies incorporating Raman microspectroscopy would further enhance the characterization of smaller microplastic particles [40].

## 5. Conclusions

This study provides evidence of microplastic presence in raw milk produced by smallholder dairy farms in Northeastern Thailand. A total of 97 microplastic particles were identified across 80 raw milk samples collected from both hand-milked and bulk tank milk. The majority of detected MPs were fiber-shaped and yellow in color, with most particles distributed within the smallest size range of 0.05–0.15 mm. FT-IR spectroscopy analysis identified Polydimethylsiloxane as the most common polymer, followed by a semi-synthetic composite material, consisting of Elastane. These findings suggest that the MPs detected in raw milk may originate from both synthetic and semi-synthetic materials associated with dairy production practices and materials in contact with milk, while also indicating potential internal exposure of dairy cows. The consistent detection of MPs in both hand-milked and bulk tank milk highlights the importance of considering animal-level exposure and milk handling processes as critical points for understanding microplastic presence in raw milk within dairy production systems.

Although this study did not evaluate toxicological effects or human exposure, the presence of MPs along the dairy production chain highlights the need for improved material management and hygiene practices at the farm level. Further studies incorporating exposure assessment and toxicological evaluation are required before potential implications for animal, environmental, or human health within a One Health framework can be determined. Additionally, enhanced farm management practices are essential, including minimizing plastic usage, improving equipment maintenance, and strengthening waste disposal protocols in dairy production. Further research is needed to trace MP contamination pathways, assess the potential physiological effects in cattle, and evaluate long-term implications for consumers. Establishing evidence-based guidelines for microplastic monitoring in livestock farming systems will be crucial for safeguarding public health and ensuring the sustainability of dairy production.

## Figures and Tables

**Table 1:** Characteristics of smallholder dairy farms and numbers of milking cows included in the study.

Farm ID	Number of Milking Cows	Number of Hand-Milked Raw Milk Sample Collection	Number of MPs Positive Hand-Milked Raw Milk Samples (Percentage)	Number of Bulk Tank Milk Sample Collection	Number of MPs Positive Bulk Tank Milk Samples (Percentage)
**A**	40	20	11 (55%)	1	1 (100%)
**B**	16	7	4 (57.14%)	1	1 (100%)
**C**	6	1	0 (0%)	1	0 (0%)
**D**	20	4	2 (50%)	1	1 (100%)
**E**	16	5	3 (60%)	1	1 (100%)
**F**	18	6	3 (50%)	1	1 (100%)
**G**	15	5	4 (80%)	1	1 (100%)
**H**	19	6	1 (16.67%)	1	0 (0%)
**I**	17	6	0 (0%)	1	1 (100%)
**J**	25	10	1 (10%)	1	1 (100%)
**Total**	192	70	29 (41.42%)	10	8 (80%)

**Table 2:** Distribution of microplastic particles across different sample types.

Farm	Hand-Milked Raw Milk (Particles, 70 Samples)	Mean MPs Concentration in Hand-Milked Raw Milk (Particles/mL)	Bulk Tank Milk (Particles, 10 Samples)	Mean MPs Concentration in Bulk Tank Milk (Particles/mL)
**A**	30	0.1	3	0.2
**B**	11	0.1	6	0.4
**C**	0	0	0	0
**D**	8	0.13	4	0.27
**E**	3	0.04	1	0.07
**F**	4	0.04	4	0.27
**G**	10	0.13	4	0.27
**H**	2	0.02	0	0
**I**	0	0	1	0.07
**J**	2	0.013	4	0.27
Mean ± SD (95% CI)	7.00 ± 9.20 (0.4–13.6)	0.057 ± 0.052 (0.020–0.095)	3.80 ± 1.30 (2.2–5.4)	0.182 ± 0.138 (0.084–0.280)
Total	70		27	

**Table 3:** Morphology, Color, and Size Distribution of Microplastics in Hand-Milked and Bulk Tank Raw Milk.

Category	Subcategory	Hand Milk *n* (%)	Bulk Milk *n* (%)	Total *n* (%)
**Morphology**	Fiber	39 (56.0)	12 (44.0)	51 (52.6)
	Fragment	26 (37.0)	11 (40.0)	37 (38.1)
	Microbead	5 (7.0)	3 (11.0)	8 (8.2)
	Film	0 (0.0)	1 (4.0)	1 (1.0)
	**Subtotal**	**70 (100)**	**27 (100)**	**97 (100)**
**Color**	Yellow	26 (37.0)	4 (15.0)	30 (30.9)
	White	19 (27.0)	6 (22.0)	25 (25.8)
	Black	9 (13.0)	12 (45.0)	21 (21.6)
	Blue	9 (13.0)	2 (7.0)	11 (11.3)
	Red	3 (4.0)	0 (0.0)	3 (3.1)
	Brown	2 (3.0)	3 (11.0)	5 (5.2)
	Green	2 (3.0)	0 (0.0)	2 (2.1)
	**Subtotal**	**70 (100)**	**27 (100)**	**97 (100)**
**Size (mm)**	0.05–0.15	70 (100)	10 (37)	80 (82.5)
	0.15–0.5	0 (0.0)	11 (40.74)	11 (11.3)
	>0.5–1	0 (0.0)	4 (14.82)	4 (4.1)
	>1–2	0 (0.0)	2 (7.4)	2 (2.1)
	**Total (pieces)**	**70 (100)**	**27 (100)**	**97 (100)**

## Data Availability

The data supporting the findings of the study are available within the article. Further inquiries can be directed to the corresponding author.
